# Evaluating the Effects of Nanosilica on Mechanical and Tribological Properties of Polyvinyl Alcohol/Polyacrylamide Polymer Composites for Artificial Cartilage from an Atomic Level

**DOI:** 10.3390/polym11010076

**Published:** 2019-01-06

**Authors:** Qinghua Wei, Yanen Wang, Yiwen Rao, Anguo Jiang, Kun Zhang, Tingli Lu, Xiongbiao Chen

**Affiliations:** 1 Department of Industry Engineering, College of Mechanical Engineering, Northwestern Polytechnical University, Xi’an 710072, China; weiqinghua@nwpu.edu.cn (Q.W.); raoyw_stars@163.com (Y.R.); jagfrank@163.com (A.J.); npu_zk@sina.com (K.Z.); 2 Department of Mechanical Engineering, College of Engineering, University of Saskatchewan, Saskatoon, SK S7N 5A9, Canada; 3 Key Laboratory for Space Bioscience & Biotechnology, School of Life Sciences, Northwestern Polytechnical University, Xi’an 710072, China; lutinglixinxin@nwpu.edu.cn

**Keywords:** Nano-silica, polymer composites, mechanical properties, tribological properties, reinforce mechanism, molecular dynamics

## Abstract

Due to the superior performances of nanosilica particles, this research has been designed to study their effects on the mechanical and trigological properties of a PVA/PAM polymer composite by a molecular dynamics simulation method. To realize the research objectives mentioned above, the molecular models of amorphous cells and sandwiched friction models for pure polyvinyl alcohol (PVA)/polyacrylamide (PAM) (component weight ratio is 1:1) and PVA/PAM/nanosilica (component weight ratio is 5.75:5.75:1) polymer composites were constructed and simulated, respectively. The simulation results of the mechanical properties show increases about 31.6% in the bulk modulus, 53.1% in the shear modulus, and 50.1% in the Young’s modulus by incorporating a nanosilica particle into a pure PVA/PAM polymer composite. Meanwhile, the changes in Cauchy pressure, B/G ratio, and Poisson’s ratio values indicate that incorporating a nanosilica particle into pure PVA/PAM weakened the ductility of the composite. Incorporating a nanosilica particle into a pure PVA/PAM composite also showed a decrease about 28.2% in the abrasion rates and relative concentration distributions of polymer molecules in the final friction models. Additionally, the binding energy and the pair correlation functions between a nanosilica particle and the polymer chains in a cubic cell demonstrate that incorporating nanosilica into PVA/PAM polymer composites improves the internal binding strength between different components through the forming hydrogen bonds. As a result, the mechanical and tribological properties of PVA/PAM polymer composites can be enhanced by incorporating nanosilica particles.

## 1. Introduction

Due to the special structure, which includes a high specific surface area and owning an abundant number of hydroxyl groups on the surface, nanosilica presents some unique physical and chemical properties [1]. Dispersing the nanosilica in polymer materials can obviously improve their mechanical properties as well as others such as their thermodynamic properties, chemical stability, corrosion resistance properties, friction resistance, and biocompatibility [2,3,4]. As a result, it has attracted tremendous attentions from both academic and engineering communities, and many previous studies on nanosilica show that it has extraordinarily outstanding mechanical and friction resistance properties. For instance, Fallah [5] and Dil [6] also studied the effects of nanosilica on the mechanical properties of poly(lactic acid)/poly(butylene adipate-co-terephthalate) (PLA/PBAT) blends and polypropylene (PP), and their results showed that the addition of nanosilica in polymers could improve the mechanical properties of polymers. Zhaohong et al. [7] conducted an experimental study to explore the friction and wear behaviors of polymer/nanosilica composites by adding nanosilica fillers. The research revealed that the polymer composites exhibited a better tribological behavior at a nanosilica content of less than 3%. Moreover, Pattnaik et al. [8] prepared a kind of bone tissue scaffold from chitosan, nanosilica, and zirconia by a freeze-drying technique; they found that the scaffold possessed a porous nature with pore dimensions suitable for cell infiltration, and is helpful for the proliferation of osteoblast cells.

Therefore, adding nanosilica particles into polymer biomaterials is a promising method for the preparation of artificial cartilage, which can not only promote cell proliferation in a polymer matrix, but also improve mechanical and tribological properties. Although a large number of research studies involving the mechanical and tribological properties of polymer/nanosilica has been conducted [6,7,9], most of them have been performed by the traditional experimental methods, and few works have been dedicated from an atomic level due to the limitations of space and time. 

Meanwhile, with the development of computer technology, the molecular dynamics (MD) simulation method is generally considered a very useful complementary tool to experimental studies on mechanical and tribological properties [10,11,12]. For instance, MD simulations were conducted to develop a tribology model by building a molecular layer structure for providing an understanding of the improved mechanical and tribological properties of the polymer composites by introduction carbon nanotube or graphene as reinforcements [10,13]. Ta et al. [14] investigated the tribological, structural, and rheological properties of thin film hexadecane confined between different iron and iron oxide surfaces by the molecular dynamics simulation method. Jing et al. [15] applied molecular dynamics simulation to study the effect of defects including vacancy and Stone–Wales defects on the Young’s modulus of graphene sheets. Besides, in our pervious works [16,17], MD simulation was also successfully performed to investigate the mechanical properties of polymer composites. 

Herein, the MD simulation method was employed to evaluate the effects of nanosilica on the mechanical and tribological properties of polyvinyl alcohol (PVA)/polyacrylamide (PAM) polymer composites. The reinforce mechanism of nanosilica in PVA/PAM blend polymer composite was also revealed from an atomic level. The molecular structures, effects of nanosilica on the mechanical and tribological performance of the PVA/PAM composite, and its reinforcement mechanism are of great interest, and are expected to guide the further development and optimization of biomaterials in artificial cartilage.

## 2. Modeling and Method

### 2.1. Amorphous Cell

**Figure 1:**
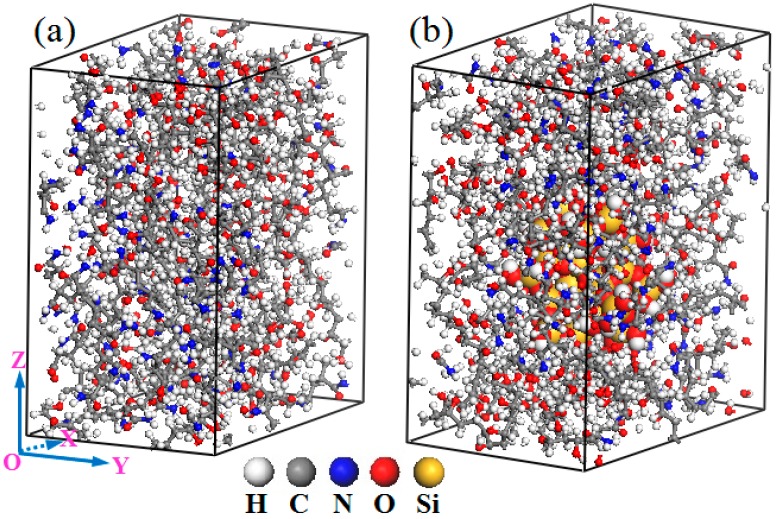
Equilibrated amorphous cells of pure polyvinyl alcohol (PVA)/ polyacrylamide (PAM) and PVA/PAM/nanosilica polymer composites.

An appropriate molecular chain length in polymers is critically important to the model development, which determines the rationality of the models and the accuracy of the calculation results. In this research, a PVA molecular chain with 50 repeat units and a PAM molecular chain with 31 repeat units were constructed as per our previous studies [17,18]. Following, according to the crystal structure of a-quartz (space group P3321) adopted from the Cambridge Structural Database, a spherical silica nanoparticle with a radius of 6 Å was constructed. The unsaturated boundary effect was avoided by adding hydrogen atoms to the unsaturated oxygen atoms and the silicon atoms of the silica particle surface. Finally, to investigate the effects of nanosilica particles on the properties of the PVA/PAM blend composite, here, two orthorhombic molecular models of pure PVA/PAM blend polymer matrix (component ratio is 50:50) and PVA/PAM/nanosilica composite (component ratio is 46:46:8) were constructed. Their final equilibrium models with same a and b parameters in sizes of 31.53 × 20.06 × 35.62 Å and 31.53 × 20.06 × 36.75 Å are shown in Figure 1. For the molecular model of the pure PVA/PAM composite (Figure 1a), it only contains four PVA molecular chains and four PAM molecular chains; the final density is 1.32 g/cm^3^. For the molecular model of the PVA/PAM/nanosilica composite (Figure 1b), it also contains four PVA molecular chains and four PAM molecular chains. Furthermore, to prevent any finite size effect of nanocomposites and account for any particle effect at the bulk level, only one nanosilica nanoparticle was embedded at the center of the blend unit cell, and the periodic boundary condition was used. The incorporation of silica results in a final density of the PVA/PAM/silica composite of 1.40 g/cm^3^. Notice that all of the models in this research were constructed by employing the Materials Studio software (Accelrys Inc., San Diego, CA, USA) [19].

### 2.2. Friction Model

**Figure 2:**
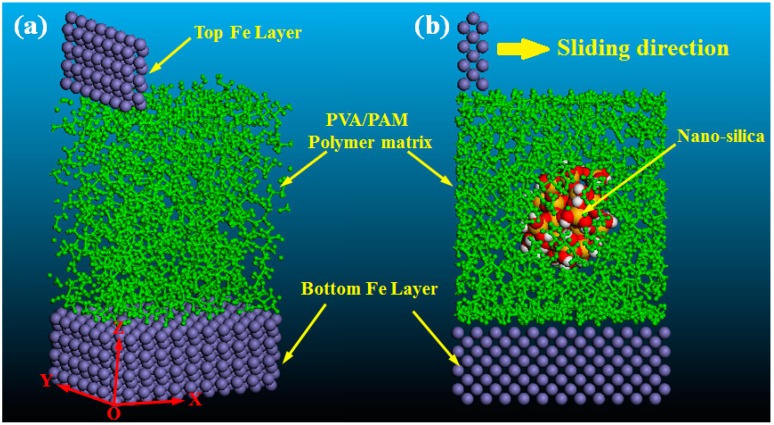
Molecular models of the friction models: (**a**) pure PVA/PAM polymer matrix; (**b**) PVA/PAM/nanosilica composites. In the models, the green denotes the polymer (PVA and PAM), the purple presents the iron, and the spherical particle denotes the nanosilica particle.

To investigate the effect of nanosilica on the tribological properties of the PVA/PAM blend composites, two friction models for the tribological processes were constructed, respectively. The initial friction models were shown in Figure 2, and the periodic boundary condition was applied in shear and transversal directions. In these two friction models, the Fe atoms layer with dimensions of 2.87 × 20.06 × 11.47 Å and 31.53 × 20.06 × 11.47 Å were constructed and used as the top and bottom frictional surfaces, respectively. The amorphous cell models of pure PVA/PAM and PVA/PAM/silica composites that were constructed in Section 2.1 were applied as the frictional materials and sandwiched between two Fe atom layers. Moreover, to better distinguish the components in the friction models, all of the polymer molecular chains were represented in green.

### 2.3. Simulation Process

For the amorphous cell, firstly, a geometry optimization was performed on the initial models to obtain the lowest energy state. The geometry optimization was performed by utilizing the smart minimization method, and the convergence tolerance was applied using customized quality with an energy convergence of 1 × 10^−4^ kcal/mol and displacement of 5 × 10^−5^ Å. The number of iterations was set as 5000. To provide further equilibration, a five-cycle anneal process was then followed under the Canonical ensemble (NVT) from 298 K to 598 K, and then cooled back at intervals of 50 K. The time step was one fs, and the number of dynamics steps per ramp was set as 1000. Secondly, the MD simulation was performed on the equilibrium systems under the Isothermal-isobaric ensemble (NPT) (P = 1 bar, T = 298 k) until the density and energy of the system no longer changed. The simulation time of this process was mainly determined by the size of the system, and here, the simulation time was 50 ps. Lastly, an additional MD simulation under the NVT ensemble (T = 298 K) was carried out on the above systems for 30 ps, and the trajectory frames of the last 20 ps were used for mechanical properties analysis.

For the friction model, firstly, a same geometry optimization and annealing process as above were performed on the initial friction models of pure PVA/PAM and PVA/PAM/nanosilica composites. Following, the composites of two friction models were fixed on the bottom Fe layer to avoid the sliding. Finally, a shear loading was applied to the top Fe layer by providing it with a sliding velocity of 0.2 Å/ps along the X direction under the NVT ensemble (T = 298 K). A uniform normal loading of 0.12 GPa was applied only on the top Fe layer, and the simulation time was 300 ps. During the friction process, the atom trajectories of friction models were recorded to investigate the tribological properties of composites. 

It is noted that all of the MD simulations in this work were performed with the COMPASS (Condensed-Phase Optimized Molecular Potentials for Atomistic Simulation Studies) force field [20], and this force field can accurately predict the structural, conformational, and thermophysical properties of a broad range of molecules and polymers [21]. It has been successfully employed to study the mechanical properties [16,22,23] and tribological properties [10,13,24] of polymers. Besides, in all of the MD simulations, the Anderson thermostat and barostat [25] were used to maintain the temperature and pressure for all of the NVT and NPT MD simulations. The Coulomb and van der Waals long-range non-bonding interactions were handled using the standard Ewald [26] and atom-based [27] summation methods, respectively. In addition, non-bonding interactions were truncated at 1.25 nm. A time step of one fs was used, and the coordinates of models were recorded every 500 time steps.

## 3. Results and Discussion

### 3.1. Mechanical Properties

In this research, the elastic constants *C_ij_* of the polymer composites were computed via the strain constant method [28] using MD simulations. The blend composites in this research are all assumed to be the isotropic material, so the bulk modulus *B*, shear modulus *G*, Young’s modulus *E*, and Poisson’s ratio *v* of the polymer composite models were determined by using the Voigt–Reuss–Hill (VRH) averaging method [29]. For the Voigt bounds, the modulus of *B* and *G* are calculated by the following equations:

(1) $$B_{V}=\frac{1}{3}(C_{11}+2C_{12}),\;G_{V}=\frac{1}{5}(C_{11}-C_{12}+3C_{44})$$

For the Reuss bounds, the modulus of B and G are express as:

(2) $$B_{R}=B_{V}=\frac{1}{3}(C_{11}+2C_{12}),\;G_{R}=\frac{5(C_{11}-C_{12})C_{44}}{4C_{44}+3(C_{11}-C_{12})}$$

Following, the Hill values are obtained by averaging the results obtained by the Voigt and Reuss methods, and they are represented by:

(3) $$B_{H}=\frac{1}{2}(B_{V}+B_{R}),\;G_{H}=\frac{1}{2}(G_{V}+G_{R})$$

Finally, the Young’s modulus *E* and Poisson’s ratio *v* can be calculated by using the Hill’s bulk modulus (*B_H_*) and shear modulus (*G_H_*), and their equations are given as:

(4) $$E=\frac{9B_{H}G_{H}}{3B_{H}+G_{H}},\;v=\frac{3B_{H}-2G_{H}}{2(3B_{H}+G_{H})}$$

According to the above principles, the elastic constants and moduli were calculated at a temperature of 298 K, which are shown in Table 1.

To our knowledge, the bulk modulus *B* of a material is a measurement of its resistance ability to volume change [30]. The calculated bulk modulus of pure PVA/PAM and PVA/PAM/silica composites are 6.73 GPa and 8.86 GPa (Table 1), which means that the PVA/PAM/silica composite has a stronger resistance ability to volume change, with an increase of about 31.6% in bulk modulus. The shear modulus *G* can reflect the resistance ability to reversible deformations upon shear stress [31]. Compared with the pure PVA/PAM, the shear modulus of the PVA/PAM/silica composite increases about 53.1% from 2.88 GPa to 4.41 GPa, this demonstrates that the PVA/PAM/silica composite has a better resistance ability to shear deformation. The stiffness of a material is determined by the Young’s modulus, with a larger value indicating higher stiffness [32]. It can be seen from the results in Table 1 that the stiffness increases 50.1% from 7.56 GPa to 11.35 GPa. Therefore, the bulk modulus, shear modulus, and Young’s modulus all can be obviously improved by incorporating nanosilica into pure PVA/PAM.

**Figure 3:**
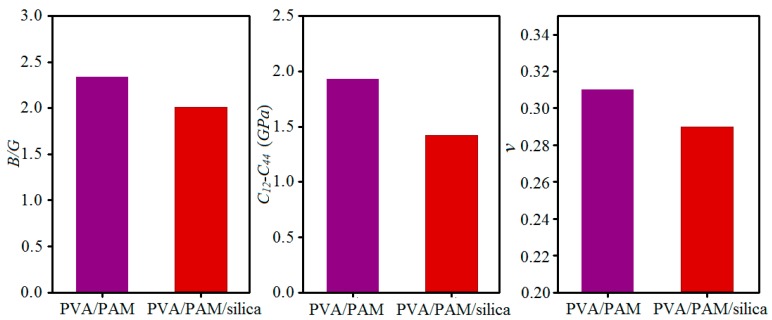
Histograms of the ratios of bulk modulus to shear modulus (*B/G*), Cauchy pressure parameter (*C*_12_–*C*_44_), and Poisson’s ratio (*v*) for pure PVA/PAM and PVA/PAM/silica composites.

Furthermore, to further evaluate the effects of nanosilica on the ductile and brittle properties of composites, here, the bulk modulus to shear modulus ratio (*B/G*), Cauchy pressure parameter ratio (*C*_12_–*C*_44_), and the Poisson’s ratio (*v*) were also calculated, which are shown in Figure 3. Cauchy pressure is often used to evaluate the ductility of a material; a more positive Cauchy pressure corresponds to a better ductility [33]. The results in Figure 3 show that the introduction of nanosilica decreases the Cauchy pressure value of pure PVA/PAM, but both the Cauchy pressure values are positive, suggesting that the PVA/PAM/silica composite generally has a good ductility, although it is less than that of pure PVA/PAM, and the incorporation of nanosilica weakens the ductility of the pure PVA/PAM composite. The ratio of the bulk modulus to shear modulus can also predict the brittle and ductile behavior of a material [34]. A high *B/G* ratio is associated with ductility, whereas a low value corresponds to a brittle nature. The critical value that separates ductile and brittle materials is around 1.75 [31]. The *B/G* ratio values in Figure 3 indicate that adding nanosilica into the PVA/PAM composite decreases the value of the *B/G* ratio; however, both of the *B/G* ratios for the two composites are larger than 1.75, demonstrating that both of them are ductile, and nanosilica weakens the ductility to a certain extent. This conclusion shows a good agreement with the Cauchy pressure results. Besides, the Poisson’s ratio of a material can also reflect the resistance ability to deformation [30], and its value is always within the range of −1 to 0.5. The smaller Poisson’s ratio leads to the material having more brittle behavior, which is consistent with the Cauchy pressure and *B/G* ratio. Therefore, the ductility of the composite could be weakened to a certain extent by incorporating nanosilica into pure PVA/PAM.

### 3.2. Tribological Properties

**Figure 4:**
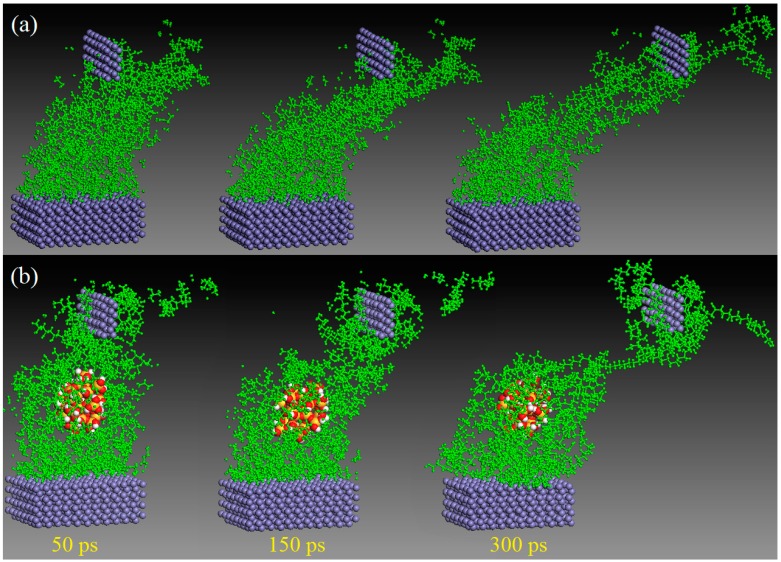
Snapshots of the friction processes of (**a**) the pure PVA/PAM and (**b**) PVA/PAM/silica composites subjected to shear loading by the top Fe layer at different time in molecular dynamics (MD) simulation.

To investigate the effect of nanosilica on the tribological properties of the PVA/PAM composite, two initial friction models of pure PVA/PAM and PVA/PAM/silica composites sandwiched between Fe layers were constructed, respectively (Figure 2). A constant sliding velocity of 0.2 Å/ps along the X direction was employed on the top Fe layer to realize the friction process. Figure 4 shows the friction processes at different simulation times for the pure PVA/PAM and PVA/PAM/silica composites. In this research, the abrasion rate was used to evaluate the tribological properties of the composites, which can be given by the ratio of the numbers of the worn atoms to the total atoms of the composite. It can be calculated by the following equation:

(5) $$Abrasion\;rate=\frac{N_{remove}}{N_{total}}\times 100\%$$

where *N_remove_* represents the number of the worn atoms that escape from the main composite, and *N_total_* denotes the number of the total atoms contained in the initial composite. To determine the abrasion rates of these two composites, the trajectories of all of the atoms in the friction models were calculated and recorded during the shear loading MD simulations. The abrasion rates of the final moment friction models (300 ps) in MD simulation were calculated and are shown in Table 2.

From the calculated results in Table 2, we can know the abrasion rates of pure PVA/PAM and PVA/PAM/silica sheared with a same sliding velocity were 39.7% and 28.5%, respectively. The abrasion rate decreases by about 37.3% after incorporating nanosilica particle into a pure PVA/PAM composite. This result can also be obtained by observing the snapshots of the friction processes of two composites in Figure 4. Due to the introduction of a nanosilica particle, the polymer chains in the PVA/PAM/silica composite are inclined to be absorbed on the surface of the nanosilica particle. This absorption behavior maintains a stronger structure and greatly decreases the abrasion rate of the composite.

**Figure 5:**
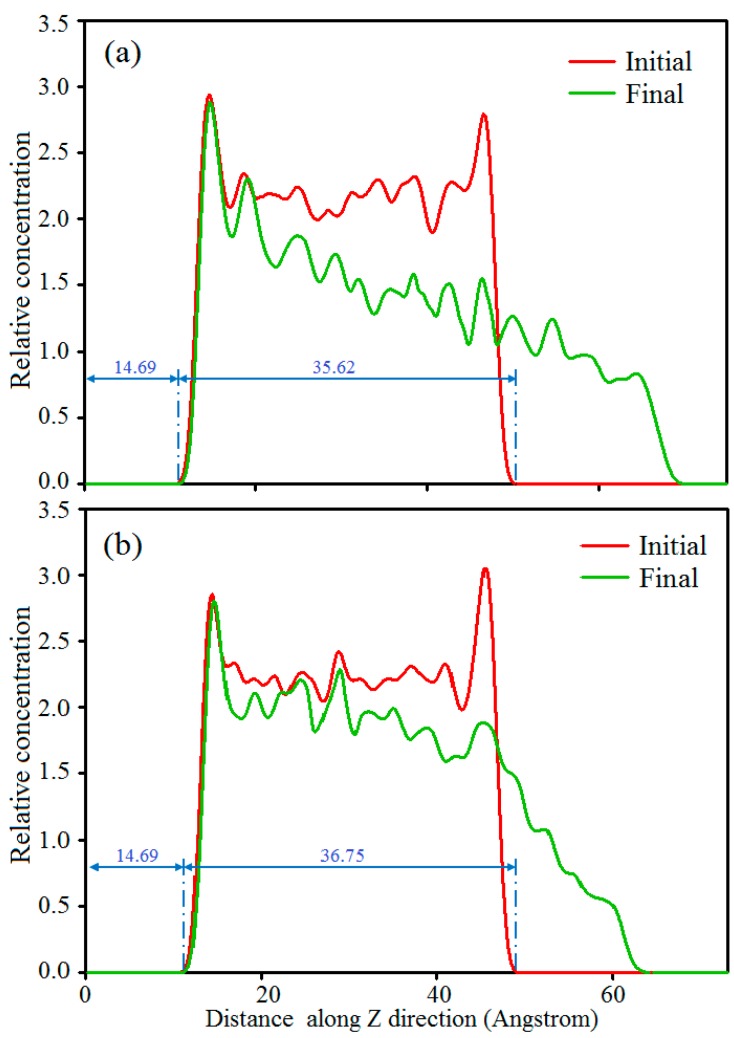
Relative concentration distributions of polymer molecules along the Z direction for (**a**) the pure PVA/PAM and (**b**) PVA/PAM/silica composites in the initial (0 ps) and final (300 ps) moments of the friction models.

Moreover, to further verify the accuracy of the results calculated above, here, the relative concentration distributions of polymer molecules along the Z direction of the initial and final moment friction models were analyzed, which are shown in Figure 5. Compared with the initial moment friction model, the change degree of the relative concentration distributions of the polymers in the final moment friction model can determine the abrasion of the composites. A more obvious change in the relative concentration distribution indicates a greater abrasion appearing on the composite material. It can be seen from the relative concentration distributions of polymer molecules for two friction models in Figure 5 that the relative concentration distributions of polymer molecules for pure PVA/PAM has a greater change than in the PVA/PAM/silica composite, which means that pure PVA/PAM possesses a greater abrasion rate. This conclusion shows a good agreement with the abrasion rate result calculated above. Therefore, incorporating nanosilica can obviously improve the shear deformation resisting ability of the PVA/PAM blend composite.

### 3.3. Preparation of Blend Membranes and their Abrasion Tests

According to the simulation models constructed above, 10 g of PVA (17-99, M_w_ 80,000) and 10 g of PAM (Analytical reagent, M_w_ 200,000) purchased from Guizhou Chemical Reagents Factory (Tongren, China) were mixed together and dissolved in 200 g of distilled water at 90 °C under continuous stirring to obtain a PVA/PAM aqueous solution. Following, the obtained solution was divided into three equal parts, and the required amount of nanosilica microspheres with diameters of 10–15 nm (99% purity) obtained from Tianjin Fu Chen Chemical Reagents Factory (Beichen, Tianjin, China) was added into the PVA/PAM solution by stirring at 50 °C until the homogeneous blends were obtained. The obtained blend solution was cast onto a polytetrafluoroethylene (PTFE) plate and dried in the vacuum oven at the temperature of 35 °C for 36 h, so that the water in blend was removed. Here, the thickness of the blend membranes were controlled by a copper spacer. Finally, the membranes (thickness = 4 mm) with different concentrations of nanosilica (0%, 4%, 8 wt %) were peeled off from the plate and cut into the same samples.

**Figure 6:**
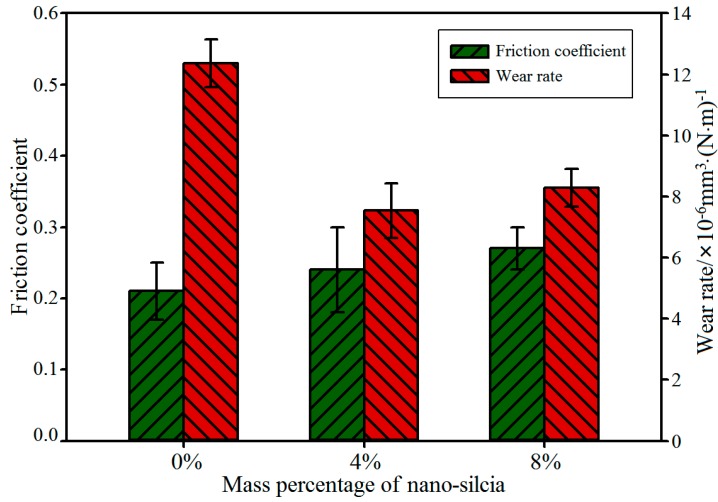
Tribological properties of PVA/PAM/nanosilica blend composites with different nanosilica content.

After that, the tribological properties of the membranes were tested by a friction and wear testing machine (MRH-1) under room temperature. During testing, the payload was set to 10 N, and the linear speed was set as 100 mm/s. After a 10-minute dry friction test, the surface morphologies of the membrane samples were observed by SEM, while the wear properties of the composites were evaluated by the volume wear rate (*K*), and its expression is as follows:

(6) $$K=\frac{\Delta m}{\rho NL}(mm^{3}/N\cdot m)$$

where Δm stands for the wear quality of the membrane samples, ρ represents the density, *N* is the payload, and *L* is the sliding friction distance. Each test was repeated three times, and the average value was calculated as the last volume wear rate; these results are shown in Figure 6.

**Figure 7:**
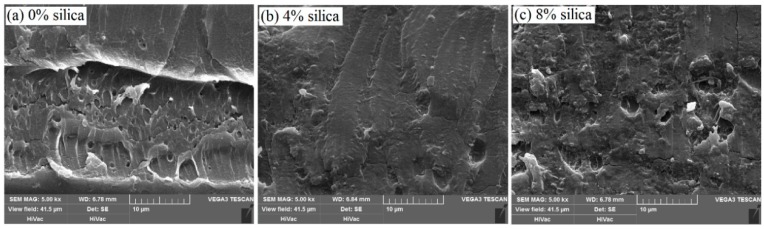
SEM images of the wear surface of PVA/PAM/nanosilica with different nanosilica contents at magnifications of 5000×: (**a**) pure PVA/PAM composite; (**b**) PVA/PAM/4% silica composite; and (**c**) PVA/PAM/8% silica composite.

From the wear test results in Figure 6, we can know that the friction coefficients of composites filled with nanosilica are larger than that of pure PVA/PAM composites, and the friction coefficient of the sample increases with the addition of nanosilica. The main reason for this phenomenon is attributed to the surface roughness that resulted from the addition of nanosilica microspheres. The calculated results of the volume wear rates show the volume wear rate decreases with the incorporation of nanosilica, and at a 4 wt % concentration of nanosilica, the wear rate of the composite sample is the smallest; it reaches 7.84 × 10^−6^ mm^3^·(N·m)^−1^, which is a decrease of about 35.8% compared with the pure PVA/PAM composite. Meanwhile, the wear rate of the composite with 8 wt % silica is larger than the composite with 4 wt % silica. The same phenomenon also can be observed from the SEM photographs of the wear surface morphology (Figure 7); as shown in Figure 7a, a deep material peeling trace appears on the friction contact surface of the pure PVA/PAM composite, which indicates that the abrasion resistance of the pure PVA/PAM composite is very poor. Compared with the pure PVA/PAM composite, the wear surface is smoother, and only a slight abrasion arises on the surface of the PVA/PAM/4% silica composite (Figure 7b). It is evident that the abrasion resistance of the PVA/PAM/4% silica composite is much better than that of the pure PVA/PAM composite. As the concentration of nanosilica increases to 8 wt %, some loose particles and pits attributed to the aggregation behavior of nanosilica occur on the surface of the composite (Figure 7c). By observing and comparing the wear surface morphologies, it is evident that the abrasion resistance of the PVA/PAM/8% silica composite is better than that of the pure PVA/PAM composite, and worse than that of the PVA/PAM/4% silica composite, implying that the appropriate concentration of nanosilica filled into the PVA/PAM composite can improve its abrasion resistance, which shows a good agreement with the above simulation results and the reports in literatures [4,35]. 

### 3.4. Reinforce Mechanism

**Figure 8:**
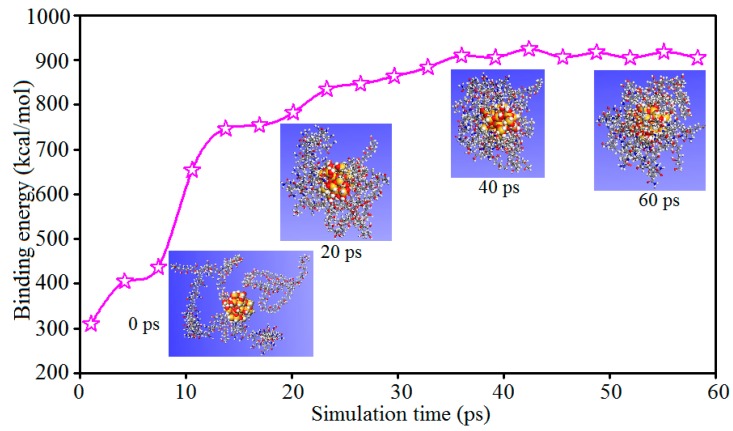
Binding energies between polymer chains and the nanosilica particle in an amorphous cell at different MD simulation times.

To elaborate the inner reason why the mechanical and tribological properties can be improved by incorporating nanosilica into a PVA/PAM polymer composite, a cubic amorphous cell of a PVA/PAM/silica molecular model was designed and constructed in which there were four PVA molecular chains, four PAM molecular chains, and one nanosilica particle. Then, the density was set as 0.6 g/cm^3^ to ensure that the polymer molecular chains in the cell had enough space to move freely. Finally, a geometry optimization and an MD simulation under the NVT ensemble were performed on the amorphous cell for 60 ps. Following that, the binding energy and pair correlation function between the nanosilica particle and the polymer chains were calculated to reveal the reinforcement mechanism. The binding energy between the nanosilica particle and polymer chains in this model can be calculated by:

(7) $$E_{bind}=-E_{\mathrm{int}er}=-(E_{total}-E_{polymer}-E_{silica})$$

where *E_total_* is the energy of the polymer/silica composite, *E_polymer_* is the energy of the polymer, and *E_silica_* is the energy of the nanosilica particle. Figure 8 shows the binding energies between the nanosilica particle and the polymer chains at different MD simulation times.

As Figure 8 indicates, the binding energy between the nanosilica particle and polymer chains in a cubic cell increases with the simulation time, and the binding energy is almost invariable at the final simulation stage. Moreover, the binding energies of the initial (0 ps) and final (60 ps) configurations of the cubic cell were estimated to be 310.2 kcal/mol and 904.3 kcal/mol; there is an increase of about 191.5% in size of binding energy. The increase of binding energy leads to the adsorption behavior between the nanosilica particle and polymer chains, which means that the polymer chains in the cell tend to wrap the nanosilica particle due to their strong interactions. From the snapshots of polymer chains at different times in the adsorption process (Figure 8), the adsorption behavior between polymer chains and the nanosilica particle can also be clearly observed. The morphologies of polymer chains change from being initially randomly scattered to finally wrapping around the nanosilica particle. That is to say, the incorporation of nanosilica improves the internal binding strength between the different components, which is consistent with the mechanical and tribological properties of the composite.

**Figure 9:**
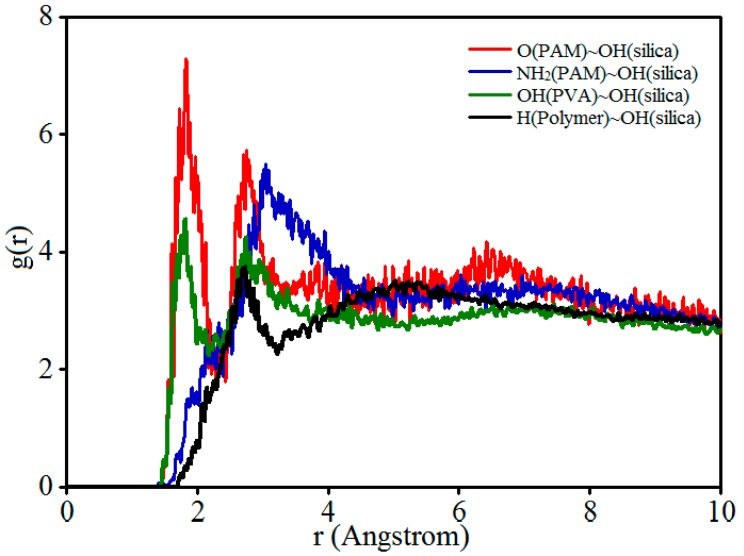
Pair correlation function (PCF) g(r) values of functional groups and polar atoms that exert stronger interaction forces in the models.

To further determine the interaction mechanism, the pair correlation functions (PCF) between polymer chains and nanosilica were studied by analyzing the final configuration (60 ps) of the amorphous cell. Here, the polymer chains that surrounded the nanosilica particle were used to analyze; Figure 9 shows the PCF g(r) values of the functional groups and polar atoms that exert stronger interaction forces in the models.

In these PCFs, OH(PVA) denotes the hydroxyl groups (–OH) of the PVA molecular chain, the oxygen of the carbonyl groups (–C=O), and the amino groups (–NH_2_) of the PAM molecular chain, which were marked as O(PAM) and NH_2_(PAM), respectively. Moreover, the rest of the hydrogen atoms directly connecting with the carbon atoms of polymers were marked as H(polymer). For the nanosilica particle, the hydroxyl groups (–OH) on the nanosilica particle surface were represented as OH(silica). 

In general, intermolecular interactions involve hydrogen bonding and van der Waals forces. A peak value below 3.5 Å indicates chemical bonds and hydrogen bonds, and values higher than 3.5 Å indicate van der Waals interactions [16,36]. As shown in Figure 9, for all of the PCFs, there is a peak value appearing at r = 1.8~3.1 Å within the scope of the hydrogen bonding interaction, predicting that the functional groups, polar atoms, and hydrogen atoms of the polymer chains interact with the hydroxyl groups on the silica surface mainly by strong hydrogen bonding. This conclusion is in agreement with the result reported by Zhang et al. [37]. Moreover, by analyzing the first peak values size of PCFs, the strength of hydrogen bond formation can be compared; the larger the peak value corresponds to the stronger interaction [38]. Obviously, the strongest hydrogen bonding interaction occurs between the oxygen atoms in the carbonyl groups of PAM (O(PAM)) and the hydroxyl groups of the nanosilica (OH(silica)), and the weakest hydrogen bonding interaction appears between the hydrogen atoms of polymer (H(polymer)) and the hydroxyl groups of the nanosilica (OH(silica)). By comparing the first peak values of the PCFs in Figure 9, the strength of each type of hydrogen bond can be determined, which has the order of O(PAM)…OH(silica) > NH_2_(PAM)…OH(silica) > OH(PVA)…OH(silica) > H(Polymer)…OH(silica). That’s to say, the wrapping behavior of the polymer chains on the surface of the nanosilica particle is mainly attributed to the strong hydrogen bonding between the function groups, the polar atoms of the polymer chains, and the hydroxyl groups on the silica surface. These computational results are in good agreement with the reported experimental results of the FTIR spectra in the literature [39]. According to the polymer deformation theory [40], the closer molecular chain entanglement, the smaller the molecular chain movement space, resulting in the enhancement of the ability to resist deformation. So, the incorporation of nanosilica improves the internal binding strength between different components by the forming hydrogen bonds, which is the root reason for the improvement of the mechanical and tribological properties of the composite.

## 4. Conclusions

In this research, the amorphous cells and friction models of pure PVA/PAM and PVA/PAM/nanosilica polymer composites were respectively constructed to investigate the effects of a nanosilica particle on the mechanical and tribological properties of polymer composites from an atomic level. The calculated results of the mechanical properties show that there are increases of about 31.6% in the bulk modulus, 53.1% in the shear modulus, and 50.1% in the Young’s modulus by incorporating a nanosilica particle into a pure PVA/PAM polymer composite. Meanwhile, the Cauchy pressure, *B/G* ratio, Poisson’s ratio values decrease with the incorporation of nanosilica, which indicates that the ductility of the composite could be weakened by incorporating nanosilica into pure PVA/PAM. Moreover, the calculated abrasion rates results show a decrease of about 37.3% by incorporating a nanosilica particle into a pure PVA/PAM composite, which is consistent with the analysis result of the relative concentration distributions of polymer molecules in the final friction models. Finally, the reinforce mechanism of nanosilica in the PVA/PAM polymer composite was revealed in terms of the binding energy and the pair correlation functions between the nanosilica particle and the polymer chains in a cubic cell. The results indicate that the incorporation of nanosilica improves the internal binding strength between different components by the forming hydrogen bonds, which is the root reason for the improvement of the mechanical and tribological properties of the composite.

## Figures and Tables

**Table 1:** The calculated elastic constants *C_ij_* and different engineering moduli of pure PVA/PAM and PVA/PAM/silica composites (GPa).

System	*C*_11_	*C*_12_	*C*_44_	*B_V_*	*G_V_*	*B_R_*	*G_R_*	*B_H_*	*G_H_*	*E*	*v*
PVA/PAM	10.48	4.86	2.93	6.73	2.88	6.73	2.88	6.73	2.88	7.56	0.31
PVA/PAM/silica	15.82	5.38	3.96	8.86	4.46	8.86	4.36	8.86	4.41	11.35	0.29

**Table 2:** Abrasion rates of pure PVA/PAM and PVA/PAM/silica composites.

System	*N_total_*	*N_remain_*	*N_remove_*	*Abrasion Rate*
PVA/PAM	2656	1602	1054	39.7%
PVA/PAM/silica	2800	2102	698	24.9%
